# The relationship between four types of premature ejaculation patients and the quality of residential environment

**DOI:** 10.1186/s12610-022-00183-7

**Published:** 2023-04-13

**Authors:** Xi Liu, Tianle Zhu, Pan Gao, Jingjing Gao, Rui Gao, Hui Jiang, Xiansheng Zhang

**Affiliations:** 1 Department of Urology, The First Affiliated Hospital of Anhui Medical University, Hefei, China; 2 Department of Urology, Peking University First Hospital Institute of Urology, Peking University Andrology Center, Peking University First Hospital No, 8 Xishiku Street Xicheng District, Beijing, China

**Keywords:** Premature Ejaculation, PREQIs, Depression, Residential environment, Éjaculation précoce, PREQI, Dépression, Environnement résidentiel

## Abstract

**Background:**

We investigated the association between premature ejaculation (PE) and the quality of residential environment from a new perspective to explore the influencing factors of PE, especially in four PE subtypes. We selected 499 adult males to participate in this study from September 2021 to September 2022. The satisfaction of residential environment was assessed by the Perceived Residential Environment Quality Indicators (PREQIs) scale, the control ability over ejaculation was assessed using the premature ejaculation diagnostic tool (PEDT), and their depression was assessed using the self-rating depression scale (SDS).

**Results:**

The Architectural and Town-planning Spaces (ATS), Green Spaces (GS), and Commercial Services (CS) of PE patients (*N* = 346) were compared with those of control group (*N* = 153), showed a significant difference (*p* < .05), for PE patients, the score of ATS was 44.30 ± 12.38, the score of GS was 18.60 ± 6.24, and the score of CS was 20.82 ± 8.20; for control group, which were 40.46 ± 16.21, 20.69 ± 5.71 and 22.90 ± 7.03 respectively. After age was taken into account, ATS had a positive correlation with PEDT score (*r* = 0.76), whereas GS and CS had a negative correlation (*r* = -0.87, -0.90); ATS had a positive correlation with SDS (*r* = 0.96), whereas GS and CS had a negative correlation (*r* = -0.74, -0.81).

**Conclusions:**

We discovered that PE patients more likely resided in high-density areas with little green space and subpar commercial services, which might have an adverse effect on their mental health. This study offered a new viewpoint about the influence of residential environment on PE.

## Résumé

**Contexte:**

Nous avons étudié l’association entre éjaculation précoce (PE) et qualité de l’environnement résidentiel sous un nouvel angle pour explorer les facteurs d’influence de la PE, en particulier dans quatre sous-types de PE. Nous avons sélectionné 499 hommes adultes pour participer à cette étude de septembre 2021 à septembre 2022. La satisfaction de l’environnement résidentiel a été évaluée par l’échelle des indicateurs de qualité perçue de l’environnement résidentiel (PREQI), la capacité de contrôle de l’éjaculation a été évaluée à l’aide de l’outil de diagnostic de la PE (PEDT) et leur dépression à l’aide de l’échelle d’auto-évaluation de la dépression (SDS).

**Résultats:**

Les espaces architecturaux et urbanistiques (ATS), les espaces verts (GS) et les services commerciaux (CS) des patients atteints de PE (*n* = 346) ont été comparés à ceux du groupe témoin (*n* = 153). Ils ont montré une différence significative (*p*<,05) ; pour les patients PE, le score de l’ATS était de 44,30±12,38, le score de GS de 18,60±6,24 et le score de CS de 20,82±8,20 ; pour le groupe témoin, ces scores étaient respectivement de 40,46±16,21, 20,69±5,71 et 22,90±7,03. Une fois l’âge pris en compte, l’ATS avait une corrélation positive avec le score PEDT (*r* = 0,76), tandis que GS et CS avaient une corrélation négative (*r* = -0,87, -0,90) ; l’ATS avait une corrélation positive avec SDS (*r* = 0,96), tandis que GS et CS avaient une corrélation négative (*r* = -0,74, -0,81).

**Conclusions:**

Nous avons découvert que les patients atteints de PE résidaient plutôt dans des zones à forte densité avec peu d’espaces verts et des services commerciaux médiocres, ce qui pourrait avoir un effet néfaste sur leur santé mentale. Cette étude a offert un nouveau point de vue sur l’influence de l’environnement résidentiel sur la PE.

## Background

Premature ejaculation (PE) is the most common ejaculatory dysfunction [1]. There were epidemiological researches showed that 5–40% of sexually active males suffered from PE [2, 3]**.** The effect on men from Middle Eastern and African countries was the smallest, on men from East Asian countries was the largest, for men from Europe, it was in the middle stage [4]. In the past, it has been challenging for academics to come to an agreement on how to more accurately characterize PE, but the below definitions were always taken into account: I. Ejaculation time; ii. Failure to regulate or extend ejaculation; iii. A series of negative effects of PE. The definition of lifelong premature ejaculation (LPE) was originally brought up by the inaugural International Society for Sexual Medicine (ISSM) Committee in 2007 [5], then LPE and acquired premature ejaculation (APE) were both defined by the second ISSM Committee in 2014 [6]. Despite this, some patients also complained of PE even though they did not meet the diagnostic criteria of LPE and APE during the clinical diagnosis and treatment process. In this instance, researchers expanded on the description of PE and introduced two new PE subtypes [7–10]: variable premature ejaculation (VPE) and subjective premature ejaculation (SPE). Subsequently the occurrence of the above two PE subtypes were verified by clinical studies in different nations (China, Turkey) [11, 12].

The development of PE was almost always influenced by psychological issues. PE was formerly considered to be a psychological illness [13], but with the evolving of this field, it had become clearer that both psychological and biological issues contributed to PE [14]. Previous studies have demonstrated that PE was linked with a wide range of psychological issues, including pervasive anxiety and depression [15–17]. According to a study in 2007 [18], PE patients exhibited lower level of sexual function and satisfaction as well as larger rate of mental and psychological suffering than men without PE. The three dimensions of personal, behavioral, and social environment were the key factors influencing mental health in the past [19], but this was not sufficient to explain the increased prevalence of mental disease. Recent research [20–22] suggested that residential environment might have a significant impact on the development of mental disease, which were through direct route like changes in psychological processes and indirect route like changes in housing conditions, congested spaces, indoor noise, and light, according to a prospective study by Evans GW in 2003 [23]. We hypothesized that the residential environment might also affect the occurrence of PE by affecting mental health. Therefore, the purpose of our study was to investigate whether there was relationship between PE and residential environment, and to explore the correlation between PE and residential environment in people who came to the hospital.

There were a lot of literature about the influence of psychological factors on PE, but there are few systematic studies on how residential environment affected PE, particularly including the four subtypes of PE. China is the largest developing country in the world [24]. With the rapid development of cities, residential environment of the residents has also changed greatly, which resulted a serious psychological influence [25]. Even though there are so many effects, the pertinent study between residential environment and mental health is still in early stage in China [26, 27]. Because that psychological factors are important factors affecting PE, so we hypothesize that the residential environment may cause PE through affecting mental health. There were studies in some European nations have verified the validity of Perceived Residential Environment Quality Indicators (PREQIs), a scale for gauging the people’s satisfaction of residential environment [28]. Additionally, a study revealed that this scale was also applicable to the Chinese men [26]. In this study, we used PREQIs to evaluate the impact of various residential environment elements on mental health. We also combined the self-rating Depression Scale (SDS) [29] and premature ejaculation diagnostic tool (PEDT) [30] scale to explore the potential impact of residential environment on PE.

## Methods

### Subjects

PE group were male patients who went to the First Affiliated Hospital of Anhui Medical University with PE as their main complaint from September 2021 to September 2022. At the same time, we gathered control group from the health examination center who underwent routine medical examinations and had no known disease. All subjects were required to meet the following conditions: (i) over 18 years old; (ii) Having a regular sexual relationship with a regular partner for at least six months ( once or twice a month for the elderly, and twice to six times per week for the young and middle-aged) [31–33]; (iii) had the ability read and write Chinese properly. In addition, another entry criterion for the PE group was that PE was the primary diagnosis. People who were on antidepressants and medications that could impair ejaculatory function were excluded from participating, as well as heavy drinkers and smokers.

### Study design

A preliminary experiment (*N* = 30) was conducted to evaluate the validity of the questionnaire. The following were the main assessment tools and factors used in this study: (i) PREQIs, (ii) SDS, (iii) PEDT, and (iv) the essential information, such as age, weight, education level, marital status, and so on. The Cronbach's alpha coefficient was employed to assess the validity of PREQIs, SDS, and PEDT, the values of internal consistency were 0.68, 0.71, and 0.76, respectively.

### Definition of PE

**Fig. 1:**
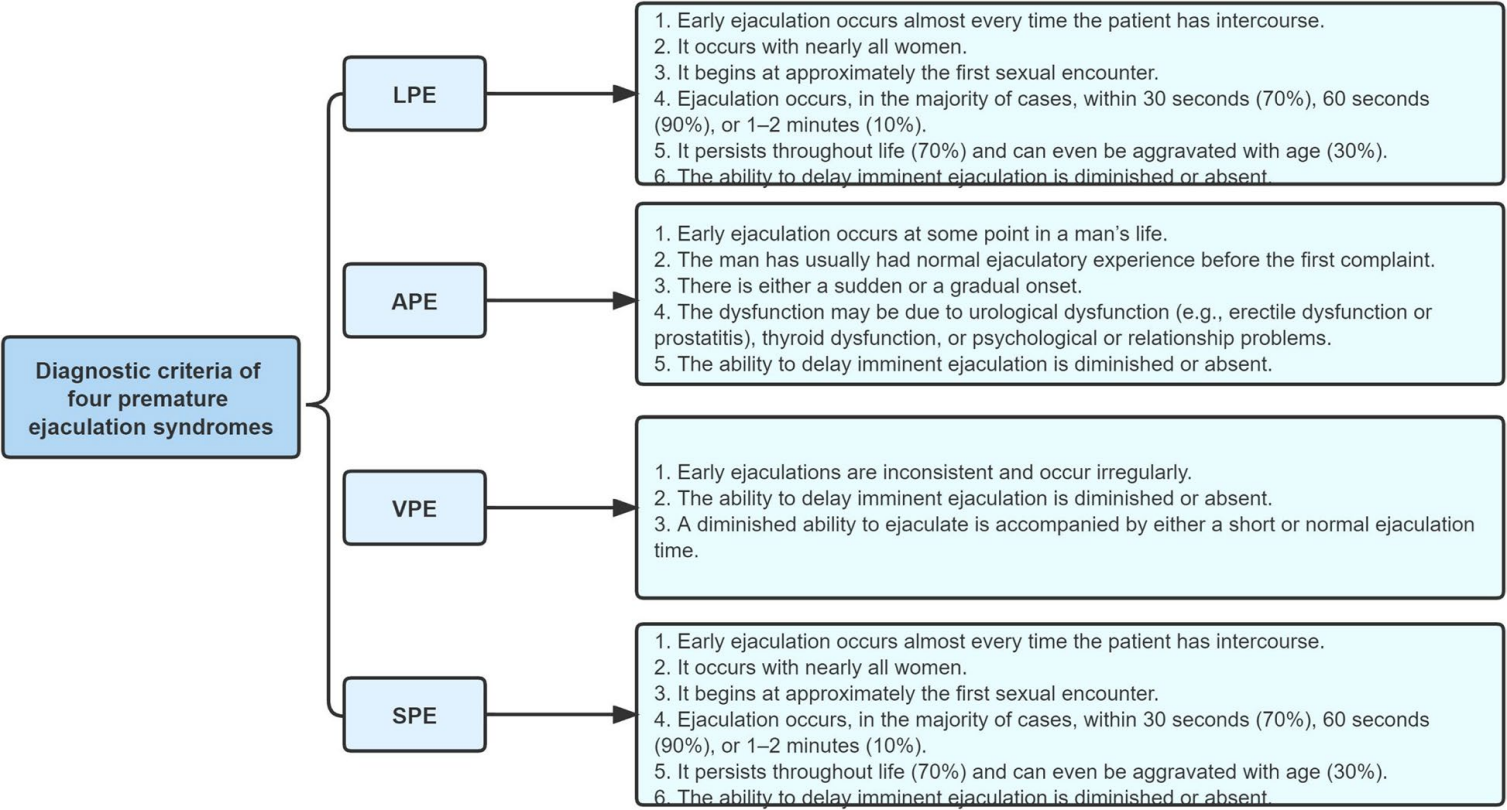
The diagnostic criteria of 4 premature ejaculation syndromes. The diagnostic criteria of 4 PE syndromes. LPE: lifelong PE, APE: acquired PE, VPE: variable PE, SPE: subjective PE

PE was defined as unsatisfied ejaculation time. The latest categorization of PE was divided into four subtypes: LPE, APE, VPE, and SPE, which was showed in Fig. 1. PEDT was used to diagnose PE frequently [30], which consists of five questions, with a 5-point Likert-type scale, from "not difficult" to “Extremely difficult” accordingly.

### Perceived Residential Environment Quality Indicators (PREQIs)

**Fig. 2:**
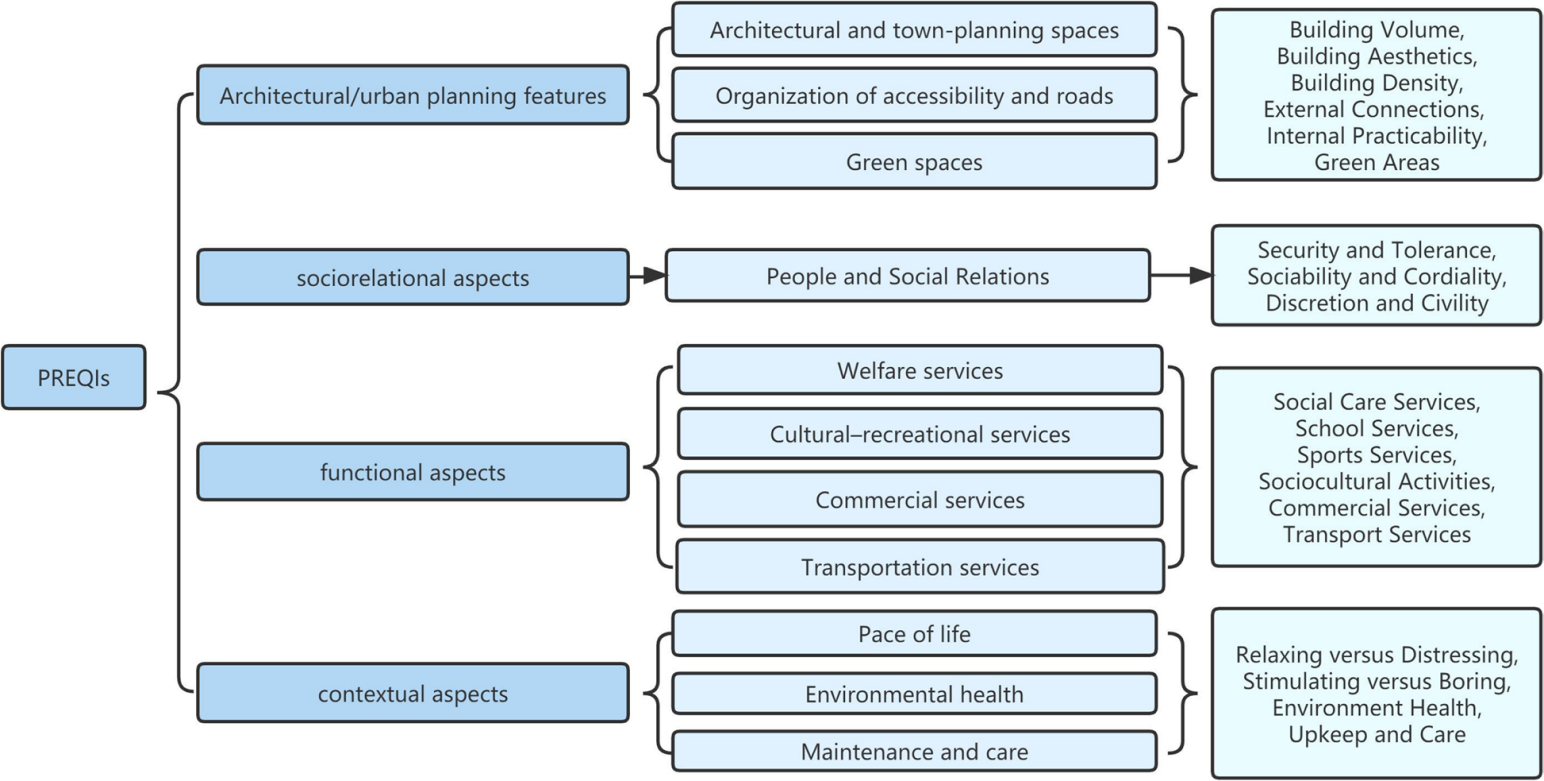
Definition of perceived residential environment quality indicators. Based on four broad dimensions and eleven specific ones, the PREQIs scale rates residents' satisfaction. PREQIs: Perceived Residential Environment Quality Indicators

The PREQIs scale evaluated people’s satisfaction of residential environment based on the four broad dimensions and eleven specific dimensions which was showed in Fig. 2. The short version of PREQIs includes 62 questions, but the full version has 140 items, both positive and negative terms are present on the scale. Responses ranged from "totally disagree" to "definitely agree" on a 5-point Likert-type scale.

### Assessment of depression

The SDS self-rating scale including 20 questions was used to evaluate mental health. The final score was calculated by the cumulative score of each item divided by 80. A score of less than 0.5 was considered no depression, whereas a score of greater than or equal to 0.5 indicated depression.

### Statistical analysis

IBM SPSS Statistics 25 (SPSS Inc., Chicago, IL, USA) was used as the analysis tool in this study. *P* < 0.05 was considered statistically significant. Descriptive statistics were used to summarize the subjects’ characteristics. The data were presented as mean ± standard deviation (SD) or percentage when appropriated. The independent t-test and analysis of variance were used for data analysis. The four subtypes of PE were compared by using one-way ANOVA. The correlation analysis was used to explore the relationship between PREQIs and PEDT scores, as well as the relationship between PREQIs and SDS scores. Since age might have an impact on PE, age was taken into account when analyzing the relationship between PREQIs and PEDT.

## Results

### Basic classification of subjects

This study included 346 male patients with PE as their primary complaint and 153 men who came to undergo routine health checkup and had no known disease. Among all PE patients, the percentages of the four PE subtypes were as follows: LPE, 8.09% (28/346), APE, 62.14% (215/346), VPE, 23.70% (82/346), and SPE, 6.07% (21/346). The detailed demographic information of subjects was shown in Table 1.

**Table 1:** Demographic information

Characteristics	Subjects who complained of PE ((*N* = 346))					Control group (*N* = 153)	*p-* value
	Total (*N* = 346)	LPE (*N* = 28)	APE (*N* = 215)	VPE (*N* = 82)	SPE (*N* = 21)		
Age, years	33.16 ± 8.24	30.25 ± 6.18	34.81 ± 9.12	31.50 ± 6.29	30.08 ± 5.90	37.40 ± 8.87	< .05
BMI, kg/m^2^	25.08 ± 3.41	26.43 ± 3.13	24.57 ± 2.84	24.52 ± 3.48	25.79 ± 2.96	24.92 ± 5.90	.96
Self-estimated IELT, minutes	2.09 ± 1.83	1.28 ± 0.21	1.52 ± 0.81	3.33 ± 1.37	5.08 ± 1.88	6.38 ± 4.03	< .001
Duration of the relationship, years	8.82 ± 4.43	9.33 ± 3.06	10.03 ± 5.76	6.92 ± 5.60	3.33 ± 1.52	12.62 ± 9.23	.09
Lifestyle, n (%)							
Smoking	138(39.89)	12(42.86)	84(39.07)	28(34.15)	14(66.67)	35(22.86)	< .001
drinking	168(48.56)	8(28.57)	105(48.84)	49(59.76)	6(28.57)	75(49.02)	.83
Exercise	185(52.60)	5(17.86)	126(58.60)	49(59.76)	5(23.81)	113(73.86)	< .001
Educational status, n (%)							.57
Primary education	74(21.39)	5(17.86)	61(28.37)	5(6.10)	3(14.29)	14(9.15)	
High school	140(40.46)	20(71.43)	81(37.67)	35(42.68)	4(19.05)	50(32.68)	
University graduate	132(38.15)	3(10.71)	73(33.95)	42(51.22)	14(66.67)	89(58.17)	
Occupational status, n (%)							.05
Student	35(10.12)	4(14.29)	21(9.77)	7(8.54)	3(14.29)	27(17.65)	
Employed	271(78.32)	19(67.86)	175(81.40)	61(74.39)	16(76.19)	110(71.90)	
Unemployed	40(11.56)	5(17.96)	19(8.83)	14(17.07)	2(9.52)	16(10.46)	
Resident, n (%)							.36
Urban	196(56.65)	6(21.43)	126(58.60)	54(65.85)	10(47.62)	101(66.01)	
Rural	150(43.35)	22(78.57)	89(41.40)	28(34.15)	11(52.38)	52(33.99)	

*PE* premature ejaculation, *LPE* lifelong PE, *APE* acquired PE, *VPE* variable PE, *SPE* subjective PE, *BMI* body mass index, *IELT* intravaginal ejaculation time

Data are presented as mean ± SD or as percentages (%)

Differences between men with and without PE were assessed by independent t-test or chi-square test as appropriate

*p*-value: differences between men with and without PE

### PREQIs and SDS scores of PE group and control group

**Fig. 3:**
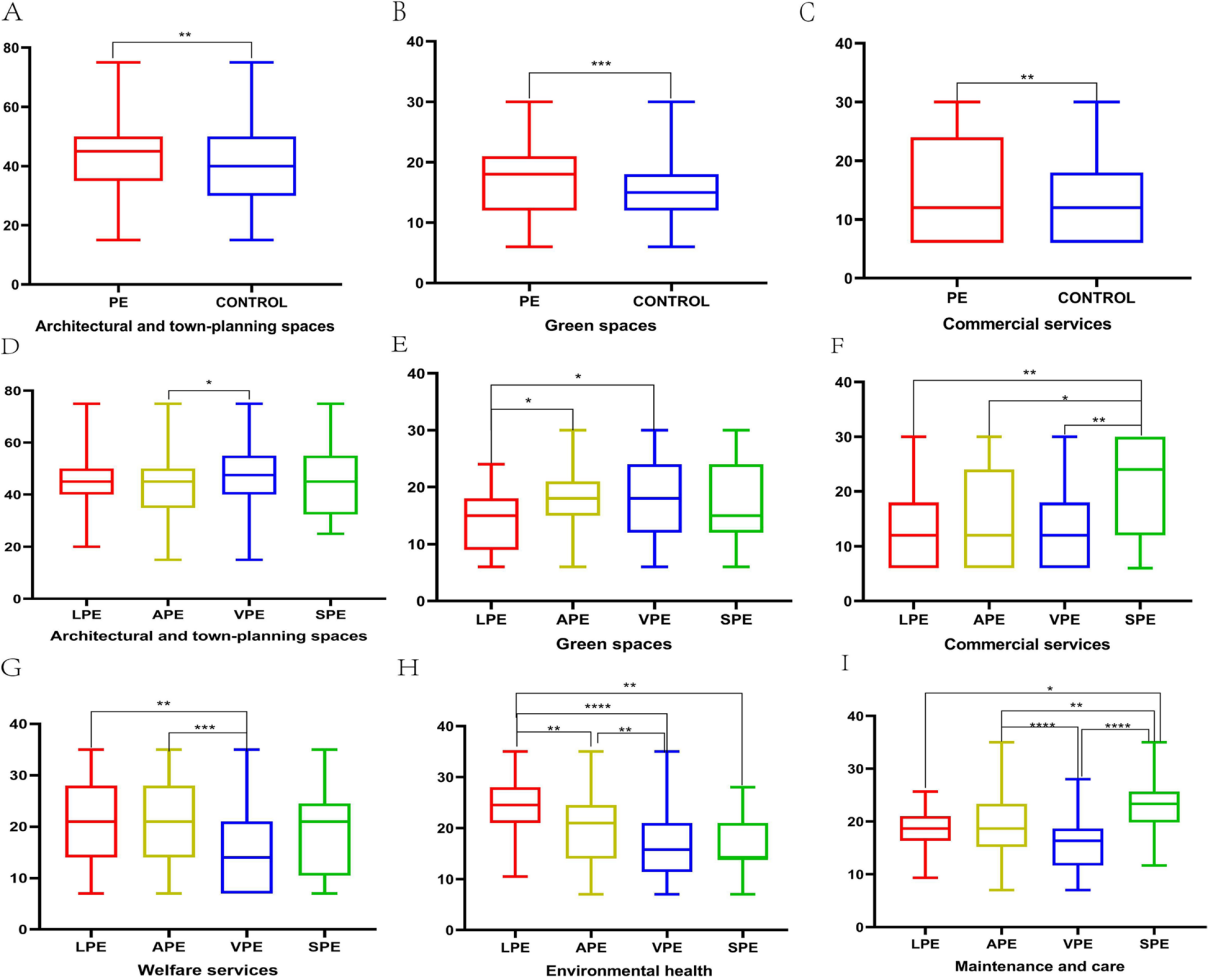
The combined box plots of data characteristics in PREQIs scale. T-test was used to analyze the difference between the premature ejaculation group and the control group, The four kinds of premature ejaculation were compared using a one-way ANOVA. **A**: The total ATS score of PE patients was greater than that of the control group (*p* < .05); **B**: The total GS score of patients was significantly lower than that of the control group (*p* < .001); **C**: The total CS score of patients was lower than that of the control group (*p* < .01); D-I: In terms of ATS, GS, CS, WS, EH and MC, male patients with different PE subtypes also have significant differences. PE: premature ejaculation, LPE: lifelong PE, APE: acquired PE, VPE: variable PE, SPE: subjective PE, ATS: Architectural and Town-planning Spaces, GS: Green Spaces, WE: Welfare services, EH: Environmental health, MC: Maintenance care. *: *p* < .05, **: *p* < .01, ***: *p* < .001

**Fig. 4:**
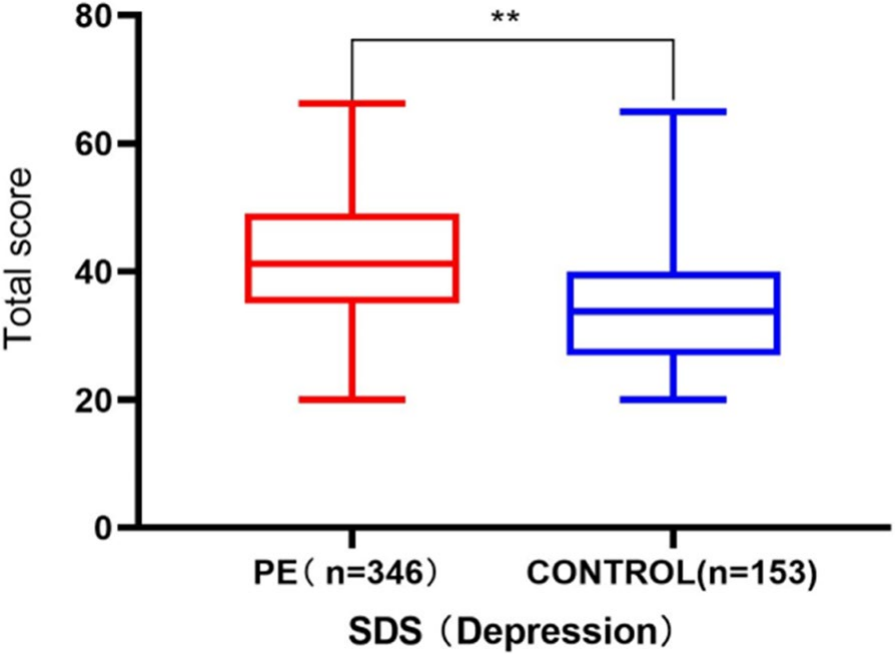
Self-rating depression scale score of premature ejaculation patients and control group. T-test was used to analyze the difference between the premature ejaculation group and the control group**.** SDS: Self-Rating Depression Scale, PE: premature ejaculation. **: *p* < .01

The quality of residential environment measured by PREQIs was listed in Table 2. In all directions of architectural/urban planning features, the Architectural and Town-planning Spaces (ATS) score of PE group was greater than that of control group (*p* < 0.05), while the Green Spaces (GS) score was significantly lower (*p* < 0.001) than that of control group, which was showed in Fig. 3 (3A, 3B). In the meanwhile that there was no statistically significant difference between the control group and PE group in the score of Organization of Accessibility and Roads (OAR). Similarly, among the four directions of functional aspects, only the commercial service (CS) score of PE group was found to be significantly lower than that of the control group (*p* < 0.01), which was shown in Fig. 3 (3c). In addition, there was no difference between the control group and PE group in the other two categories (the Sociorelational Aspects and the Contextual Aspects). The SDS score of PE group was significantly higher than that of control group (*p* < 0.01), which was showed in Table 3 and Fig. 4.

**Table 2:** Result of the perceived residential environment quality indicators

	With PE complaint	Control group						
Inventory	(*n* = 346)	(*n* = 153)	*P* -value	LPE	APE	VPE	SPE	*P -*value
Architectural/urban Planning Features:								
Architectural and Town-planning Spaces	44.30 ± 12.38	40.46 ± 16.21	< .05	45.36 ± 12.54	43.02 ± 12.37^c^	47.08 ± 11.85^b^	45.00 ± 13.13	< .05
Organization of Accessibility and Roads	26.53 ± 8.06	27.30 ± 8.07	.32	28.21 ± 9.33	26.01 ± 7.59	27.50 ± 8.50	25.71 ± 9.09	.32
Green Spaces	18.60 ± 6.24	20.69 ± 5.71	< .001	21.86 ± 5.82^b, c^	18.29 ± 5.75^a^	18.25 ± 7.13^a^	18.86 ± 7.11	< .05
Sociorelational Aspects:								
People and Social Relations	23.82 ± 6.58	22.65 ± 5.55	.05	26.52 ± 5.06	23.64 ± 6.41	23.01 ± 7.10	25.39 ± 7.19	.06
Functional Aspects:								
Welfare Services	17.86 ± 9.23	18.12 ± 8.59	.77	21.00 ± 9.53^c^	18.90 ± 9.47^c^	14.00 ± 7.53^a, b^	18.33 ± 8.42	< .0001
Cultural–recreational Services	24.74 ± 13.13	23.07 ± 11.66	.18	22.14 ± 11.01	25.07 ± 13.37	25.00 ± 13.58	23.81 ± 11.61	.71
Commercial Services	20.82 ± 8.20	22.90 ± 7.03	< .01	22.71 ± 6.60^d^	20.35 ± 8.30^d^	13.21 ± 7.67^d^	15.43 ± 8.39^a, b, c^	< .001
Transportation Services	14.23 ± 6.84	13.46 ± 6.08	.23	15.00 ± 6.94	13.99 ± 6.78	13.99 ± 6.98	16.67 ± 6.77	.34
Contextual Aspects:								
Pace of Life	30.27 ± 15.07	29.55 ± 13.32	.61	38.50 ± 13.88^b^	28.64 ± 14.63^a^	31.95 ± 15.71	29.33 ± 15.29	< .05
Environmental Health	19.12 ± 6.76	18.90 ± 6.16	.73	23.75 ± 6.13^b, c, d^	19.63 ± 6.69^a, c^	16.83 ± 6.44^a, b^	16.83 ± 5.60^a^	< .0001
Maintenance and Care	18.75 ± 5.84	19.32 ± 5.57	.31	18.67 ± 4.02^d^	19.40 ± 5.86^c, d^	15.89 ± 5.08^b, d^	23.67 ± 5.68^a, b, c^	< .0001

*PREQIs* Perceived Residential Environment Quality Indicators, *PE* premature ejaculation, *LPE* lifelong PE, *APE* acquired PE, *VPE* variable PE, *SPE* subjective PE

Data are presented as mean ± SD or as percentages (%)

Differences between men with and without PE were assessed by independent t-test as appropriate

Difference among 4 PE syndromes was assessed by one-way analysis of variance

*p*-value: differences between men with and without PE

^a^Significant difference compared with LPE

^b^Significant difference compared with APE

^c^Significant difference compared with VPE

^d^Significant difference compared with SPE

**Table 3:** Result of the self-Rating depression scale and index of PE in all subjects

	With PE complaint	Control group					
Inventory	(*n* = 346)	(*n* = 153)	*P*-value	LPE	APE	VPE	SPE
Index of PE							
Total Score (Q1-Q5)	18.46 ± 3.33	-	-	19.01 ± 4.69	19.10 ± 3.14	16.75 ± 3.36	18.15 ± 1.26
Control over Ejaculation (Q1, Q2, Q3)	11.00 ± 2.17	-	-	11.64 ± 3.37	11.45 ± 2.06	10.25 ± 1.86	9.83 ± 1.47
Distress about PE (Q4)	3.74 ± 1.07	-	-	4.51 ± 0.58	3.81 ± 1.05	3.25 ± 1.14	4.16 ± 0.89
Sexual Satisfaction (Q5)	3.72 ± 1.11	-	-	2.86 ± 1.29	3.84 ± 1.10	3.25 ± 1.06	4.16 ± 0.98
Index of SDS							
Depression Score	41.63 ± 11.01	35.28 ± 10.32	< .01	46.88 ± 16.02	39.96 ± 11.11	42.60 ± 9.59	47.92 ± 8.04

*SDS* Self-Rating Depression Scale, *PE* premature ejaculation, *LPE* lifelong PE, *APE* acquired PE, *VPE* variable PE, *SPE* subjective PE

Data are presented as mean ± SD or as percentages (%)

Differences between men with and without PE were assessed by independent t-test as appropriate

Difference among 4 PE syndromes was assessed by one-way analysis of variance

p-value: differences between men with and without PE

^#^: Q1-Q5 were the five questions, used to diagnose PE according to the Premature Ejaculation Diagnostic Tool (PEDT)

### The PREQIs and PEDT scores of four PE subtypes

The results revealed significant variations among male patients with various PE subtypes, which were showed in Fig. 3 (3D-I) and Table 2. Patients with the VPE subtype had the highest average ATS score (47.08 ± 11.85), whereas APE subtype had the lowest average score (43.02 ± 12.37) in the four PE subtypes. Additionally, the average GS score of patients with the LPE subtype (21.86 ± 5.82) was higher than that of VPE subtype (18.25 ± 7.13). Patients with the LPE subtype had the highest average CS score (22.71 ± 6.60), whereas VPE subtype was the lowest (13.21 ± 7.67). Furthermore, APE patients had a higher overall PEDT score than the other three PE subtypes. Patients with APE subtype demonstrated worse ejaculation control, lower sexual satisfaction, and higher concern about PE disease compared to the other three PE subtypes (Table 3).

### Relationship between ATS, GS, CS and PEDT scores in patients with PE complaints

There were correlations between ATS, GS, CS and PEDT score in PE patients (Table 4). The ATS score was positively correlated with the PEDT total and subdomain scores (Total scores: Adjusted *r* = 0.76, *p* < 0.05; Items of control over ejaculation: Adjusted *r* = 0.89, *p* < 0.001; Item of distress about PE: Adjusted *r* = 0.86, *p* < 0.01; Item of sexual satisfaction: Adjusted *r* = 0.90, *p* < 0.01) after age was taken into account. The total and subdomain scores of the PEDT were negatively correlated with GS (Total scores: Adjusted *r* = -0.87, *p* < 0.05; Items of control over ejaculation: Adjusted *r* = -0.89, *p* < 0.01; Item of distress about PE: Adjusted *r* = -0.97, *p* < 0.001; Item of sexual satisfaction: Adjusted *r* = -0.85, *p* < 0.01). The comparison of the CS with PEDT scores were negatively correlated (Total scores: Adjusted *r* = -0.90, *p* < 0.01; Items of Control over ejaculation: Adjusted *r* = -0.87, *p* < 0.01; Item of distress about PE: Adjusted *r* = -0.91, *p* < 0.01; Item of Sexual Satisfaction: Adjusted *r* = -0.92, *p* < 0.01).

**Table 4:** The correlation analysis of PREQIs with PE index and SDS

	PEDT								SDS	
	Total Scores		Control over Ejaculation		Distress about PE		Sexual Satisfaction		Total Scores	
Inventory	Adjusted r	*p*	Adjusted r	*p*	Adjusted r	*p*	Adjusted r	*p*	Adjusted r	*p*
PREQIs: Architectural/urban Planning Features										
Architectural and Town-planning Spaces	0.76	< .05	0.89	< .001	0.86	< .01	0.90	< .01	0.96	< .001
Green Spaces	-0.87	< .05	-0.89	< .01	-0.97	< .001	-0.85	< .01	-0.74	< .05
functional Aspects										
Commercial Services	-0.90	< .01	-0.87	< .01	-0.91	< .01	-0.92	< .01	-0.81	< .01

*PREQIs* Perceived Residential Environment Quality Indicators, *PEDT* premature ejaculation diagnostic tool, *PE* premature ejaculation, *SDS* Self-Rating Depression Scale

Correlations between outcomes of the PREQIs and PEDT scores were assessed using partial correlations

^₱^: Adjusted R-Squared offset the effect of the number of samples

The ATS score was positively correlated with the PEDT total score and subdomain score. The total and subdomain scores of the PEDT were negatively correlated with GS and CS. The SDS total score was positively correlated with ATS score and negatively correlated with GS and CS

### Relationship between ATS, GS, CS and SDS scores in patients with PE complaints

Patients with PE symptoms had correlations between their ATS, GS, CS and SDS scores (Table 4). The ATS score was positively correlated with the SDS score (Adjusted *r* = 0.96, *p* < 0.001) after age was taken into account. The SDS score were negatively correlated with GS (Adjusted *r* = -0.74, *p* < 0.05). The comparison of CS score and SDS score revealed similar negative associations (Adjusted *r* = -0.81, *p* < 0.001).

## Discussion

The impact of residential environment on premature ejaculation (PE), particularly in four PE subtypes, has few been systematically evaluated. According to the latest classification, diverse PE subtypes has different pathophysiological causes and therapeutic choices [7, 8]. To further explore the effect of residential environment on PE, PREQIs scale was used to evaluate the satisfaction of residential environment and SDS scale was used to evaluate the psychological status of all subjects. These results could serve as a theoretical foundation for understanding the relationship between PE syndrome and the quality of residential environment in China as well as for the individualized care of PE patients.

Those findings revealed that the ATS score of PE group was significantly higher compared to that of control group (*p* < 0.05), while the GS and CS scores were significantly lower than control group (*p* < 0.01), indicating that PE patients may live in an environment with a high population density, little living space, little greenery, and subpar commercial services. Subsequently, we conducted a more detailed analysis about the ATS, GS, and CS scores in four PE subtypes. Patients with VPE subtype had the highest ATS score, while APE subtype had the lowest, indicating that VPE patients had the highest community residential density, while APE subtype had the lowest. The residential environment of LPE patients (21.86 ± 5.82) had the highest green space, while VPE subtype (18.25 ± 7.13) had the lowest in the four PE subtypes, in the meanwhile CS score had the same outcome. In all three comparisons, VPE (considered psychogenic in nature) [34] had the lowest GS and CS scores and the highest ATS score. In the PREQIs scale, negative statements were used for ATS and positive statements were used for GS and CS, per the explanation in Bonaiuto M et al. [28]. The results of the correlation study revealed that PEDT was correlated with ATS positively (*p* < 0.01) and was correlated with GS and CS negatively (*p* < 0.01). In addition, we discovered that the SDS score of PE group (41.63 ± 11.01) was much higher than that of control group (35.28 ± 10.32), and this difference was statistically significant (*p* < 0.01). There was no statistically difference between the average SDS score of the four PE subtypes: LPE (46.88 ± 16.02), APE (39.96 ± 11.11), VPE (42.60 ± 9.59), and SPE (47.92 ± 8.04). This result was consistent with a previous study by Yang Y et al. [35], that PE patients have a high prevalence of depression.

ATS, an umbrella term for community architecture and urban planning, contains the index of building volume, aesthetics and density [36]. It goes without saying that ATS is important in community settings. ATS is one of the characteristics of urban built environments that may have an impact on people's mental health and overall well-being. A study in Hong Kong, China [27], found that while a rise in residential density was positively correlated with the prevalence of depression, an increase in livable area was associated with a lower prevalence of depression. Similar study in the United Kingdom found that crowding was linked to worse mental health [37]. It was discovered in our study that the average ATS score of PE group (44.30 ± 12.38) was statistically higher than that of control group (40.46 ± 16.21), and the difference was similarly statistically significant (*p* < 0.05) in the comparison of four PE subtypes. The greatest VPE score was 47.08 ± 11.85, while the lowest APE score was (43.02 ± 12.37). The correlation study revealed that ATS score and PEDT score showed a correlation in PE patients, there was a significant positive association between them in the overall score and subdomain scores (*p* < 0.05) based on the adjusted R value. At the same time, the SDS score of PE patients in this survey was also significantly higher than control group. The correlation study revealed that PE patients also showed a link between ATS score and SDS score, there was a significant positive association between them in the overall score (*p* < 0.001) based on the adjusted r value. As a conclusion, we assumed that ATS in residential environment may be a risk factor affecting psychogenic PE.

GS, such as parks, woodlands, street trees and vegetation cover, as a factor with increasing concern, plays a positive role in mental health [38, 39]. Low GS may be one of the causes of depression, and this may also play a role in the pathogenesis of PE. Among adults, the rates of depression and anxiety were also lower in greener environments [40, 41], which may be related to attention recovery, stress release, increased physical activity, and/or enhanced social contact [42, 43]. There was a study showed that the annual prevalence of depression was found to be lower in communities with more green Spaces within a 1 km radius [44]. This relationship was stronger for depression than for any other disease group cluster, demonstrating the importance of green spaces for mental health in clinical populations. The same results were also found in our study, with PE patients having a higher risk of depression than control group (*p* < 0.01), and control group having a higher mean GS score (20.69 ± 5.71) than PE patients (18.60 ± 6.24) (*p* < 0.001). Similarly, the GS score of LPE was the highest and APE was the lowest among the four subtypes of PE patients, which was statistically significant (*p* < 0.5). The GS score of LPE subtype patients was higher than that of control group. Therefore we speculated that external factors such as living environment may play an important role in exacerbating the development of LPE (rapid ejaculation occurs after in first sexual contact), rather than in the pathogenesis of LPE. According to the results of the correlation study, GS exhibited significant negative relationship with both the PDET (*p* < 0.05) and SDS (*p* < 0.05) scores. Therefore, we hypothesized that low GS may affect PE patients by affecting the mental health.

CS, as a measure of life satisfaction [28], could make people feel relaxed. As a subjective evaluation of the overall quality of life, life satisfaction is one of the core elements of happiness and the core construction of positive psychology, which has been paid great attention by psychologists [45]. CS is especially important when considering immobile human lives in residential isolation situations, such as during the COVID-19 pandemic. In our work, we found that the CS score of PE patients (including each subtype) was significantly lower than that of control group (*p* < 0.001), at the same time, the average CS scores of the four PE subtypes were also statistically significant (*p* < 0.001), and the correlation analysis showed that CS exhibited significant negative relationship with both the PDET (*p* < 0.01) and SDS (*p* < 0.01) scores. Therefore, CS may act as an influencing factor on the occurrence of PE.

### Limitations of the study

There were still some limitations in this study. First, the sample size was insufficient, there were fewer than 30 male patients in the two PE subtypes (LPE was 28 cases, SPE was 21 cases). Secondly, in this study, only PE was classified according to the current reference indicators and depression was not graded. We will expand the research scope, increase the number of samples to conduct more detailed stratified research in the future. Finally, only untreated patients were included in this study. future studies will include all patients and modify their treatment plans to better understand the effect of the residential environment on premature ejaculation. In the trials that follow, we will concentrate on altering the individuals' residential environment and investigating the impact of the residential environment on premature ejaculation to determine whether the residential environment regulates premature ejaculation.

## Conclusions

This was the first time to evaluate the impact of residential environment on PE systematically, especially among the four PE subtypes. In our study, people who living in less green space, poor commercial services and high residential density were more likely to have a negative impact on mental health, thus affecting the occurrence of PE indirectly. This study provides a new perspective for understanding the impact of residential environment on PE, and helps clinicians to understand the influencing factors and pathogenesis of PE from multiple perspectives.

## Data Availability

The data used during the current study are available from the corresponding author on reasonable request.

## Abbreviations

PE: Premature ejaculation
ISSM: International Society of International Medicine
LPE: Lifelong premature ejaculation
APE: Acquired premature ejaculation
VPE: Variable premature ejaculation
SPE: Subjective premature ejaculation
PREQIs: Perceived Residential Environment Quality Indicators
SDS: Self-rating Depression Scale
PEDT: Premature ejaculation diagnostic tool
ATS: Architectural and Town-planning Spaces
GS: Green Spaces
OAR: Organization of Accessibility and Roads
CS: Commercial Services
WE: Welfare services
EH: Environmental health
MC: Maintenance care
